# Genome-wide association study of eating and cooking qualities in different subpopulations of rice (*Oryza sativa* L.)

**DOI:** 10.1186/s12864-016-3000-z

**Published:** 2016-08-20

**Authors:** Feifei Xu, Jinsong Bao, Qiang He, Yong-Jin Park

**Affiliations:** 1 Department of Plant Resources, College of Industrial Science, Kongju National University, Yesan, 32439 Republic of Korea; 2 Institute of Nuclear Agricultural Sciences, College of Agriculture and Biotechnology, Zhejiang University, Huajiachi Campus, Hangzhou, 310029 China; 3 Center for crop genetic resource and breeding (CCGRB), Kongju National University, Cheonan, 31080 Republic of Korea

**Keywords:** Rice, Eating and cooking quality, Subpopulations, GWAS

## Abstract

**Background:**

Starch and protein are two major components of polished rice, and the amylose and protein contents affect eating and cooking qualities (ECQs). In the present study, genome-wide association study with high-quality re-sequencing data was performed for 10 ECQs in a panel of 227 non-glutinous rice accessions and four derived panels.

**Results:**

Population structure accounted for high phenotypic variation in three routine panels and had minor effects on subspecies-based panels. Using the mixed linear model method based on the P + K model, we detected 29, 24, 16, 17, and 29 loci that were significant for ECQ parameters in each of the five panels. Some of these loci were close to starch synthesis-related genes. Two quantitative trait loci (QTLs) (chr.9: 15417525 ~ 15474876; 17538294 ~ 18443016) for several starch paste viscosity properties detected in four panels were close to the *isoamylase 3* gene, one QTL (chr.1: 30627943 ~ 31668474) for consistency detected in three panels was close to the *starch synthase IV-1* gene. The QTL (chr.7: 1118122 ~ 1967247) for breakdown (BD), detected in the whole panel and *japonica* panel, and one QTL (chr.7: 25312126 ~ 26540950) for BD and setback (SB), detected in the whole panel and *indica* panel, may be specific gene alleles in *japonica* or *indica* panels. One previously detected QTL (chr.11: 22240707 ~ 22563596) for protein content and one new QTL (chr.5: 7756614 ~ 8042699) for many ECQ traits detected in more than two panels, may represent valuable targets for future cloning of the underlying genes.

**Conclusions:**

This study detected minor-effect QTLs affecting ECQs, and may increase our understanding of the genetic differences regulating the formation of ECQ between *indica* and *japonica* varieties.

**Electronic supplementary material:**

The online version of this article (doi:10.1186/s12864-016-3000-z) contains supplementary material, which is available to authorized users.

## Background

Improving grain quality and grain yield are among the most important objectives in rice breeding programs. Apparent amylose content (AAC) is known to affect the eating and cooking quality (ECQ) of rice [1]. However, the rapid viscosity analyser (RVA) profile can distinguish eating quality rice varieties with similar AACs by evaluating the breakdown (BD), setback (SB), and consistency (CS) viscosities [2]. In general, rice varieties with good eating quality have a BD of more than 100 rapid visco units (RVU) and an SB of less than 25 RVU [3]. Therefore, starch paste viscosities play an essential role in estimating the eating, cooking, and processing qualities of rice, and have received more and more attention in rice breeding programs [4, 5].

The *Wx* gene encodes granule bound starch synthase I (GBSSI), which is responsible for amylose synthesis. Starch paste viscosity properties are predominantly controlled by the *Wx* gene, as well as minor-effect genes with various additive effects [6–8]. The starch paste viscosity of glutinous rice differs from that of non-glutinous rice due to its lack of amylose [1]. Since GBSSI is inactive in glutinous varieties, the starch paste properties are thought to be controlled by other genes. The *pullulanase* (*PUL*) gene plays an important role in the control of peak viscosity (PV), hot paste viscosity (HPV), cold paste viscosity (CPV), BD, peak time (PeT), and pasting temperature (PTemp) in glutinous rice [4]. S*oluble starch synthase IIa* (*SSIIa*) is responsible for HPV, CPV, BD, SB, PeT, and PTemp, as well as the thermal properties of glutinous rice [9]. Polymorphisms in both *SBE1* and *SBE3* loci account for 70 % of the observed variations in HPV and CPV, and account for 40 % in both PV and CS in waxy rice [10]. Many populations derived from bi-parental crosses with similar AACs were developed to identify QTLs for starch paste viscosities and eliminate the *Wx* effects in non-glutinous accessions [7, 11]. A QTL cluster close to *SSIIa* that had major effects on the alkali spreading value (ASV), pasting time, and minor effects on gel consistency, AAC, PV, CPV, BD, and SB was identified [7]. The *branching enzyme IIb* (*BEIIb* or *SBE3*) gene cluster is responsible for PV, HPV, CPV, BD, CS, and gel consistency [7] . Another method to avoid the effects of the *Wx* gene on starch paste viscosity was performed to detect more QTLs within sub-populations of the same *Wx* background [8, 12].

Association mapping is a powerful method to connect structural genomics and phenomics in plants, provided that information on population structure and linkage disequilibrium (LD) are available [13]. Association mapping, especially candidate-gene association mapping, is commonly used to detect QTLs for starch qualities [4, 9, 14, 15]. Tian et al. [14] found that the starch synthesis-related genes (SSRGs) cooperated with each other to form a regulatory network that controls the ECQs and defines correlations among AAC, ASV, and gel consistency. Yan et al. [4] genotyped waxy rice with 17 SSRGs using 43 gene-tagged molecular markers, and found that 10 SSRGs are involved in controlling the RVA profile parameters. However, candidate-gene association mapping will miss other unknown loci, and genome-wide association studies (GWAS) are comprehensive approaches to systemically search the genome for causal genetic variations [16]. The distribution of functional alleles is strongly correlated with population structure since LD can be caused by an admixture of subpopulations, which leads to false-positive results, particularly in populations with a small number of samples [17]. An ideal sample with a subtle population structure and familial relatedness is the best panel to control for false-negatives [17]. Moreover, subdividing the diversity panel to analyze subpopulations independently using the mixed model provides a reasonable solution to lower false-positive or -negative rates [18]. For example, Huang et al. [19] chose only the *indica* landrace population to conduct GWAS for 14 agronomic traits. Huang et al. [20] collected phenotypic data of flowering time and grain-related traits for a GWAS on the *indica* or *japonica* subpopulations and the whole population, respectively.

Two subspecies of rice (*Oryza sativa* L.), *indica* and *japonica*, differ in several morphological and physiological characteristics including grain shape, shattering, apiculus hair length, leaf color, seed dormancy, cold tolerance, phenol, and sensitivity to potassium chlorate [21–23]. Moreover, *japonica* and *indica* rice varieties vary in starch physicochemical properties, which strongly influences rice cooking and processing methods for food and industrial applications [24]. The *Wx*^*a*^ allele is predominant in non-waxy *indica* cultivars with high AACs (22–29 %), whereas the *Wx*^*b*^ allele, with low AACs (12–19 %), is common to the non-waxy *japonica* variety [25–27]. The splice donor mutation is prevalent in *temperate japonica* rice but rare or absent in *indica* and *tropical japonica* rice [28]. The *indica* type *SSIIa* can convert S-type amylopectin to L-type amylopectin by elongating short chains of DP (degree of polymerization) ≤10 to form chains of DP 13–25 [24]. The *japonica* S-type amylopectin has enriched short branch chains of DP ≤10 and depleted intermediate chains of 12 ≤DP ≤24 compared with the *indica* L-type amylopectin, whereas the proportion of cluster interconnecting long chains of DP ≥25 are comparable in the two varieties [29]. Bao et al. [30] classified 56 glutinous rice varieties into two groups according to the gelatinization temperature (GT), corresponding to high GT and low GT, and found that all 15 high GT accessions belonged to *indica* rice. Despite the two genes (*Wx* and *SSIIa*) being well-studied, the exact genetic effects of other SSRGs in shaping grain quality and starch paste viscosities in different rice subpopulations remain unclear [4].

In the present study, we divided the 227 non-glutinous rice accessions into two panels and two ecotype based panels, and explored the genetics of 10 ECQ parameters in different panels and ecotypes based on genome-wide association mapping with high-quality re-sequencing data. These results will increase our understanding of the mechanism of ECQ properties in different ecotypes and how minor-effect QTLs function on ECQs.

## Results

### Phenotypic variation and contribution of population structure to phenotypic variation

**Fig. 1:**
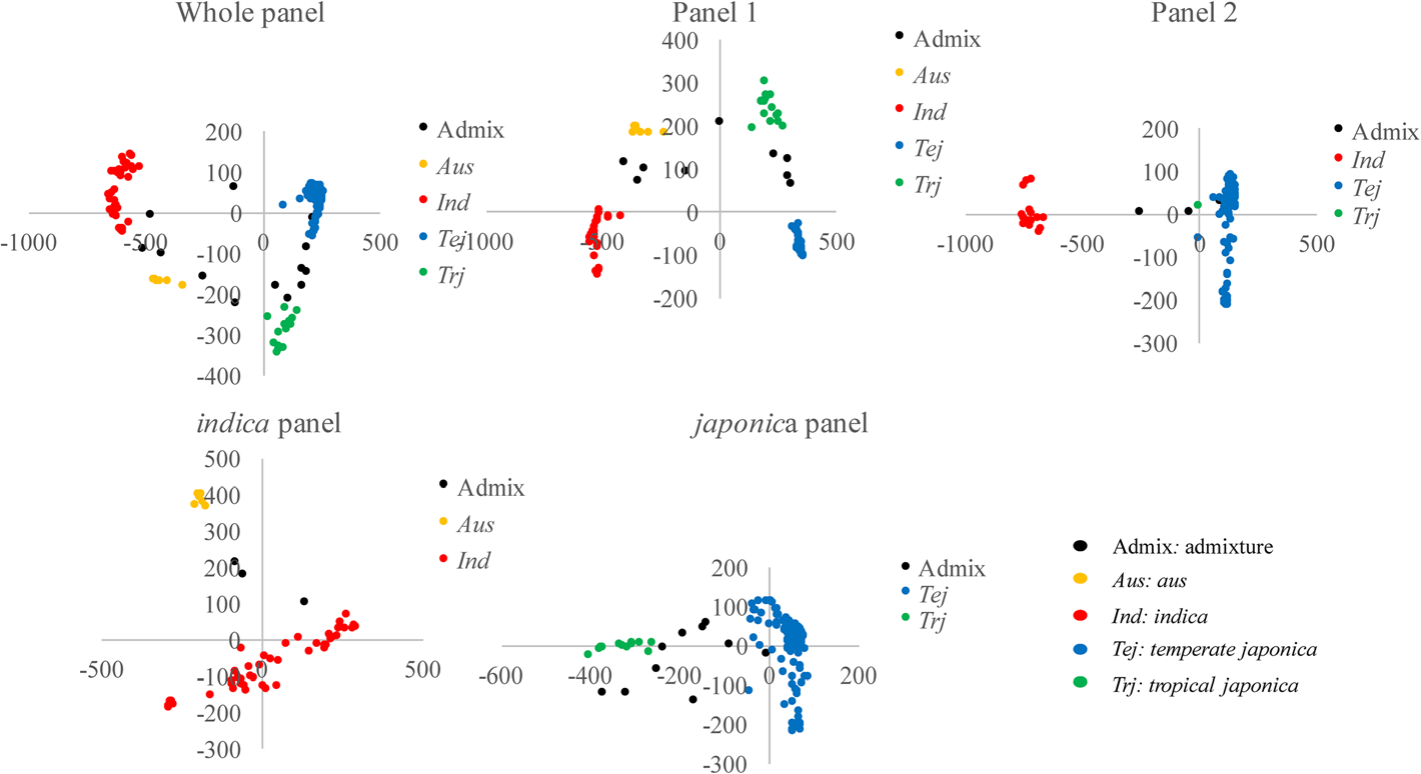
Principal component analysis (first two components) in 5 panels

**Fig. 2:**
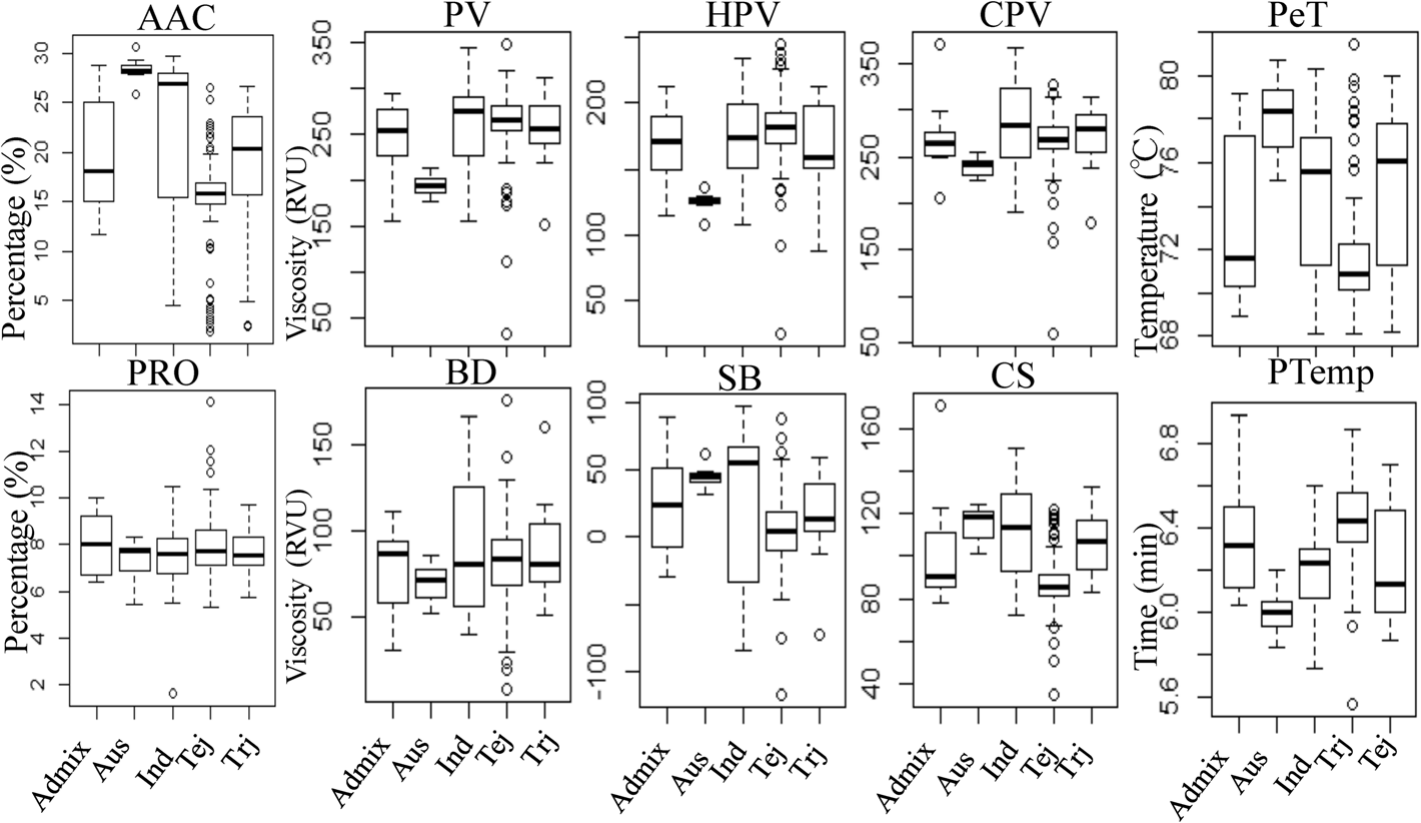
Phenotypic variation among the different ecotype groups

Population structure was evaluated using the ADMIXTURE program, which estimates individual ancestry and admixture proportions assuming that K populations exist based on a maximum-likelihood method [31]. Because rice (*Oryza sativa* L.) has two subspecies, it was not surprising that *indica* and *japonica* subpopulations could be identified. However, the *indica* subpopulation could be further divided into *indica, aus*, as well as some admixtures (admix), while the *japonica* subpopulation could be divided into *temperate japonica*, *tropical japonica*, and some admixtures (Additional file 1: Figure S1). Principal component analysis (PCA) plots of the first two components (Fig. 1) clearly differentiated rice accessions into different subpopulations, which corresponded with the population structure results (Fig. 1; Additional file 1: Figure S1). All 10 phenotypic variations in the admixture group showed moderate levels. The *aus* group had the highest AAC, PeT, SB, and CS, but the lowest PV, HPV, CPV, BD, and PTemp. The *indica* group had a wide phenotypic variation range with regards to AAC, PV, HPV, CPV, BD, SB, and CS compared to the other groups, and its AAC, PV, CPV, SB, and CS mid-values were higher than those of the *japonica* group. *Temperate japonica* had the lowest AAC, SB, CS, and PeT, and the highest PTemp. The phenotypic variation of *tropical japonica* was between that of *indica* and *temperate japonica*. However, there was no significant difference in protein content (PRO) among groups (Fig. 2).

With respect to the whole panel, a wide range in all traits was observed for AAC (10.2 ~ 30.7 %), PRO (1.6 ~ 14.1 %), PV (32.7 ~ 347.8RVU), HPV (24.2 ~ 245.3 RVU), CPV (59.6 ~ 371.5 RVU), BD (8.4 ~ 179.5 RVU), SB (−117.1 ~ 97.2 RVU), CS (28.6 ~ 170.6 RVU), PeT (3.7 ~ 6.9 min), and PTemp (68.1 ~ 81.5 °C) (Table 1). When comparing the *indica* panel with the *japonica* panel, the AAC, CPV, SB, CS, and PTemp of the *indica* panel were significantly higher than those of the *japonica* panel. The PRO, HPV, and PeT of the *indica* panel were significantly lower than those of the *japonica* panel, whereas PV and BD were similar between the two panels (Table 1). Furthermore, the AAC, PRO, SB, CS, and PTemp of Panel 1 were significantly higher than those of Panel 2. Panel 1 had lower PV, HPV, BD, and PeT than Panel 2 (Additional file 1: Table S1).

**Table 1:** Phenotypic variation in different panels and ecotype (*indica* and *japonica* panels) contribution to phenotypic variation

		AAC	PRO	PV	HPV	CPV	BD	SB	CS	PeT	PTemp
	Means ± SD^1^	18.5 ± 3.8	7.8 ± 1.3	260.2 ± 37.6	176.0 ± 27.0	271.6 ± 32.9	84.1 ± 27.3	11.4 ± 35.5	95.1 ± 15.4	6.4 ± 0.2	72.7 ± 3.0
whole	Range	10.2 ~ 30.7	1.6 ~ 14.1	32.7 ~ 347.8	24.2 ~ 245.3	59.6 ~ 371.5	8.4 ~ 179.5	−117.1 ~ 97.2	28.6 ~ 170.6	3.7 ~ 6.9	68.1 ~ 81.5
panel	CV^2^	20.7	16.0	14.5	15.4	12.1	32.4	311.0	16.2	3.1	4.1
	R square	0.429	0.030	0.100	0.122	0.084	0.029	0.078	0.365	0.277	0.224
	*P* value	<.0001	0.1547	0.0001	<.0001	0.0007	0.1689	0.0014	<.0001	<.0001	<.0001
	Means ± SD^1^	24.0 ± 5.8a	7.5 ± 1.4b	254.1 ± 42.0a	169.4 ± 30.0b	281.8 ± 42.9a	84.8 ± 37.0a	27.7 ± 51.8a	112.4 ± 20.1a	6.2 ± 0.2b	75.0 ± 3.2a
*indica*	Range	12.9 ~ 30.7	1.6 ~ 10.5	155.6 ~ 344.0	107.2 ~ 234.5	191.2 ~ 371.5	40.2 ~ 166.9	−84.5 ~ 97.2	72.5 ~ 170.6	5.7 ~ 6.6	68.1 ~ 80.7
panel	CV^2^	24.1	18.7	16.5	17.7	15.2	43.6	187.0	17.9	2.9	4.3
	R square	0.107	0.007	0.372	0.325	0.214	0.092	0.036	0.014	0.131	0.139
	*P* value	0.044	0.824	0.002	0.006	0.044	0.286	0.625	0.828	0.021	0.016
	Means ± SD^1^	16.7 ± 2.7b	7.9 ± 1.2a	262.6 ± 35.1a	179.2 ± 26.7a	267.78 ± 29.2b	83.4 ± 23.4a	5.2 ± 26.6b	88.4 ± 11.9b	6.4 ± 0.2a	71.9 ± 2.8b
*japonica*	Range	10.2 ~ 26.7	5.3 ~ 14.1	32.7 ~ 347.8	24.2 ~ 245.3	59.6 ~ 326.5	8.4 ~ 179.5	−117.1 ~ 87.5	28.6 ~ 132.5	3.7 ~ 6.9	68.1 ~ 81.5
panel	CV^2^	16.0	15.2	13.4	14.9	10.9	28.1	509.6	13.5	3.3	3.9
	R square	0.119	0.008	0.001	0.005	0.016	0.003	0.020	0.146	0.026	0.050
	*P* value	<.0001	0.537	0.937	0.698	0.287	0.774	0.195	<.0001	0.127	0.017

1. SD: standard deviation; 2. CV: coefficient of variation; 3: different letters mean significant difference between *indica* and *japonica* panels (*P* < 0.05)

Given that population structure is a main factor affecting GWAS, we evaluated phenotypic variation among different subspecies groups (*indica/japonica*) in the whole panel (Fig. 2), as well as the population structure contribution for phenotypic variation in each panel (Table 1 and Additional file 1: Table S1).

In the whole panel, population structure explained 42.9 (AAC), 10.0 (PV), 12.2 (HPV), 8.4 (CPV), 7.8 (SB), 36.5 (CS), 27.7 (PeT), and 22.4 % (PTemp) of phenotypic variation (*P* <0.01) (Table 1). However, population structure affected only two traits in the *indica* panel (PV and HPV) and two traits in the *japonica* panel (AAC and CS) at the significance level of *P* <0.01, and affected 6 traits (AAC, PV, HPV, CPV, PeT, and PTemp) in *indica* panel and 3 traits (AAC, CS, and PTemp) in *japonica* panel at significance level of *P* <0.05 (Table 1). In Panel 1, except for BD, population structure accounted for large phenotypic variation explanation (PVE), ranging from 13.8 % (PTemp) to 57.4 % (AAC) at a significance level of *P* <0.001. In Panel 2, population structure explained 37.5 (PV), 46.0 (BD), 25.7 (SB), 22.6 (CS), and 25.3 % (PeT) phenotypic variation (*P* <0.01) (Additional file 1: Table S1).

### GWAS of 10 ECQ parameters

**Fig. 3:**
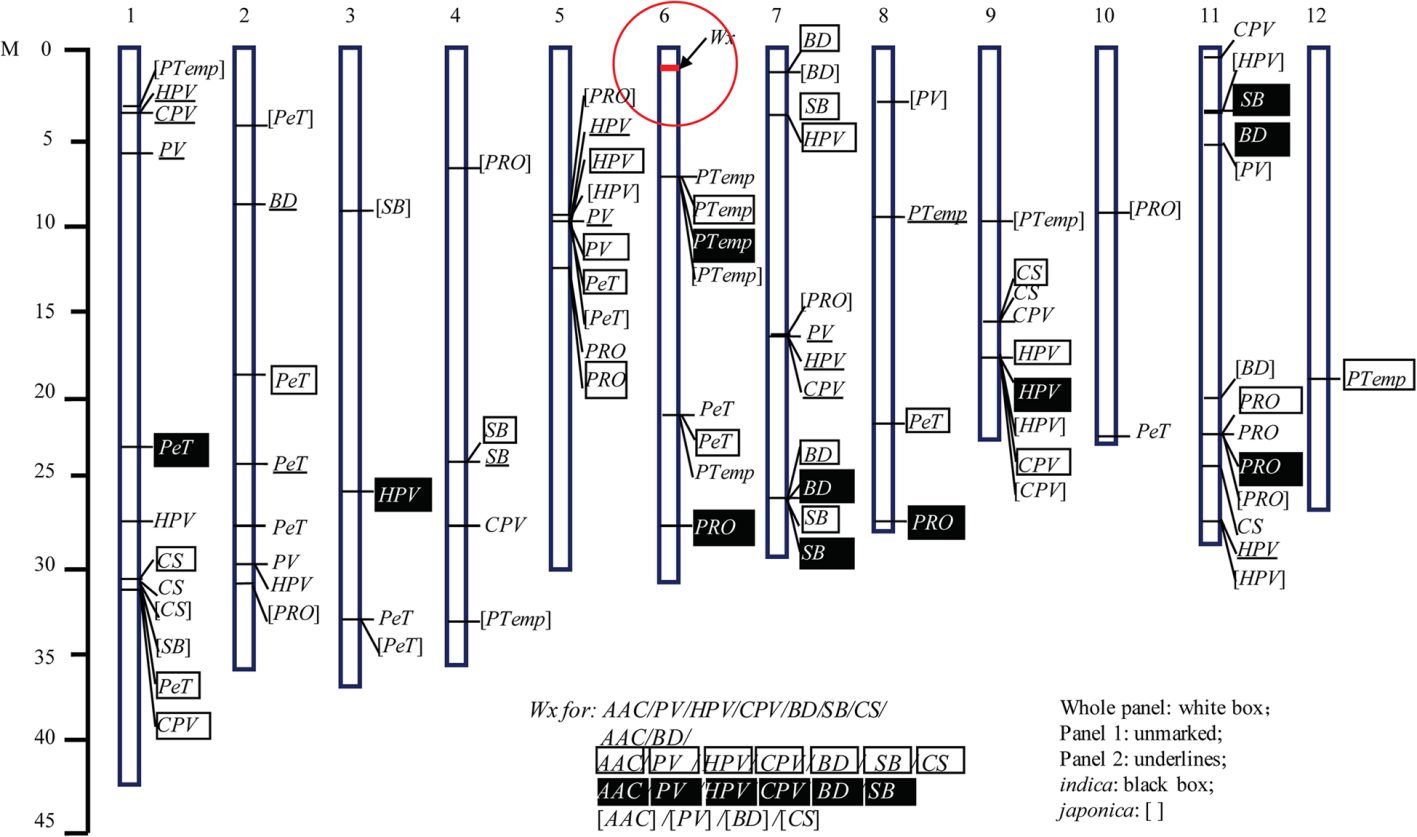
Distribution of the significant loci for ECQs in five panels (The locus in the red circle indicates the *Wx* gene for AAC, PV, HPV, CPV, BD, SB, and CS in whole panel and Panel 1, for AAC and BD in panel 2; for AAC, PV, HPV, CPV, BD, and SB in *indica* panel, for AAC, PV, BD, and CS in *japonica* panel. Loci with white box, unmarked, underlines, black box, and brackets were detected in the whole panel, Panel 1, Panel 2, *indica* and *japonica* panels, respectively.)

There were 29, 24, 16, 17, and 29 loci identified in the whole panel, Panel 1, Panel 2, *indica* panel, and *japonica* panel, respectively (Tables 2, 3, and 4, Fig. 3).

**Table 2:** Loci detected for eating and cooking qualities in the whole panel

	Chr.	Position	*P* value	MAF^a^	R square^b^
AAC	6	1765761	3.87 × 10^−18^	0.288	0.087
PRO	5	13312843	6.43 × 10^−10^	0.058	0.153
	11	22562237	2.14 × 10^−8^	0.071	0.124
PV	5	7756614	3.40 × 10^−6^	0.235	0.070
	6	1765761	3.50 × 10^−7^	0.285	0.085
HPV	5	7944999	2.25 × 10^−5^	0.071	0.067
	6	1750498	1.37 × 10^−6^	0.077	0.088
	7	3359832	9.88 × 10^−5^	0.199	0.056
	9	17538294	7.44 × 10^−5^	0.071	0.058
CPV	1	31640680	1.05 × 10^−6^	0.277	0.087
	6	1750498	6.88 × 10^−8^	0.077	0.108
	9	17539188	1.53 × 10^−6^	0.071	0.084
BD	6	1765761	3.24 × 10^−8^	0.285	0.105
	7	1967427	4.05 × 10^−6^	0.173	0.071
	7	26540950	3.34 × 10^−6^	0.093	0.073
SB	4	24293919	3.44 × 10^−7^	0.166	0.069
	6	1765761	1.69 × 10^−2^	0.285	0.140
	7	1967427	2.11 × 10^−7^	0.173	0.072
	7	25326697	5.68 × 10^−7^	0.080	0.066
CS	1	30634461	2.42 × 10^−6^	0.261	0.041
	6	1765761	8.64 × 10^−8^	0.285	0.054
	9	15474876	4.59 × 10^−6^	0.215	0.039
PeT	1	31330790	1.39 × 10^−7^	0.265	0.087
	2	18003109	6.84 × 10^−8^	0.268	0.091
	5	8042699	1.06 × 10^−6^	0.173	0.074
	6	21450052	1.67 × 10^−7^	0.058	0.085
	8	21593355	2.64 × 10^−7^	0.270	0.082
PTemp	6	7002095	1.99 × 10^−9^	0.219	0.065
	12	17937512	1.59 × 10^−6^	0.157	0.041

a: MAF: minor allele frequency; b: R square was calculated by R square of model with SNP minus R square of model without SNP

**Table 3:** Loci detected for eating and cooking qualities in Panel 1 and Panel 2

Trait			Panel1			Panel 2		
	Chr.	Position	*P* value	MAF^a^	R square^b^	*P* value	MAF^a^	R square^b^
AAC	6	1765761	5.29 × 10^−11^	0.436	0.152			
	6	1585864				9.21 × 10^−8^	0.052	0.231
	9	19310549				2.32 × 10^−7^	0.143	0.214
PRO	5	12559930	1.57 × 10^−7^	0.110	0.242			
	11	22563596	2.47 × 10^−6^	0.092	0.190			
PV	1	5102566				6.31 × 10^−5^	0.155	0.118
	2	29640874	7.77 × 10^−5^	0.136	0.118			
	5	7944999				4.15 × 10^−5^	0.086	0.125
	6	1666386	2.86 × 10^−5^	0.177	0.133			
	7	16503569				1.62 × 10^−5^	0.194	0.139
HPV	1	3541593				1.72 × 10^−5^	0.164	0.169
	1	27287504	3.51 × 10^−5^	0.082	0.127			
	2	29640874	5.22 × 10^−5^	0.136	0.121			
	5	7944999				2.88 × 10^−5^	0.086	0.159
	6	1750498	5.19 × 10^−6^	0.141	0.157			
	7	16506178				2.63 × 10^−5^	0.181	0.161
	11	27792076				6.92 × 10^−6^	0.147	0.186
CPV	1	3541593				4.78 × 10^−6^	0.164	0.178
	4	27772775	1.59 × 10^−5^	0.336	0.130			
	6	1750498	3.78 × 10^−5^	0.141	0.118			
	7	16506178				4.68 × 10^−6^	0.181	0.178
	9	15417525	2.31 × 10^−5^	0.282	0.125			
	11	192690	4.16 × 10^−5^	0.136	0.116			
BD	2	8596549				5.57 × 10^−5^	0.138	0.102
	6	1765761	1.16 × 10^−5^	0.441	0.183			
	6	1985864				9.85 × 10^−5^	0.052	0.095
SB	4	24056499				1.09 × 10^−6^	0.125	0.165
	6	1765761	2.39 × 10^−8^	0.441	0.261			
CS	1	30627943	1.98 × 10^−5^	0.214	0.111			
	6	1765761	2.41 × 10^−6^	0.441	0.138			
	9	15474876	2.19 × 10^−5^	0.277	0.110			
	11	24255295	1.96 × 10^−5^	0.118	0.111			
PeT	2	24259382				7.68 × 10^−5^	0.086	0.115
	2	27305077	1.11 × 10-^5^	0.055	0.131			
	3	33893179	1.12 × 10^−5^	0.068	0.131			
	6	21443081	8.35 × 10^−6^	0.059	0.135			
	10	23087051	9.69 × 10^−6^	0.132	0.133			
PTemp	6	6757255	1.54 × 10^−8^	0.491	0.217			
	6	21712093	1.11 × 10^−6^	0.118	0.155			
	8	9459681				4.28 × 10^−5^	0.233	0.141

a: MAF: minor allele frequency; b: R square was calculated by R square of model with SNP minus R square of model without SNP

**Table 4:** Loci detected for eating and cooking qualities in *indica* and *japonica* panels

Trait			*indica* panel			*japonica* panel		
	Chr.	Position	*P* value	MAF^a^	R square^b^	*P* value	MAF^a^	R square^b^
AAC	6	1529682	2.15 × 10^−7^	0.331	0.079			
	6	1765761				1.69 × 10^−13^	0.146	0.279
PRO	2	3118861				1.87 × 10^−5^	0.098	0.076
	4	6536977				1.37 × 10^−5^	0.055	0.079
	5	6814906				3.10 × 10^−5^	0.122	0.072
	6	2576841	2.23 × 10^−5^	0.458	-			
	7	1631971				9.32 × 10^−7^	0.073	0.102
	8	2755270	4.39 × 10^−5^	0.280	-			
	10	9032827				4.38 × 10^−6^	0.207	0.089
	11	22240707	3.60 × 10^−5^	0.093	-			
	11	22563596				2.89 × 10^−5^	0.064	0.073
PV	6	1665483	2.96 × 10^−5^	0.322	0.127			
	6	1765761				6.42 × 10^−6^	0.145	0.110
	8	2885700				6.78 × 10^−5^	0.087	0.085
	11	5061851				1.46 × 10^−5^	0.084	0.101
HPV	3	26688241	7.17 × 10^−5^	0.449	0.625			
	5	7944999				6.99 × 10^−5^	0.096	0.089
	6	1750498	1.50 × 10^−6^	0.280	0.387			
	9	18431810				6.09 × 10^−5^	0.142	0.090
	9	18729438	6.47 × 10^−5^	0.203	0.250			
	11	3161548				2.46 × 10^−5^	0.078	0.101
	11	27792076				3.84 × 10^−5^	0.229	0.096
CPV	6	1750498	1.24 × 10^−6^	0.280	0.402			
	9	18443016				1.28 × 10^−6^	0.054	0.133
BD	6	1721792	9.99 × 10^−5^	0.339	0.097			
	7	1118122				1.51 × 10^−5^	0.054	0.111
	7	26540950	6.68 × 10^−5^	0.339	0.103			
	11	3735389	4.38 × 10^−5^	0.398	0.109			
	11	20805844				1.84 × 10-^5^	0.160	0.109
SB	1	31774927				4.37 × 10^−5^	0.205	0.092
	3	8987316				2.53 × 10^−5^	0.075	0.098
	6	1617418	6.01 × 10^−6^	0.339	0.133			
	6	1765761				1.82 × 10^−8^	0.145	0.183
	7	25312126	9.18 × 10^−5^	0.297	0.095			
	11	3781133	9.23 × 10^−5^	0.356	0.095			
CS	1	31668474				5.17 × 10^−6^	0.202	0.085
	6	1759451				6.46 × 10^−7^	0.120	0.102
PeT	1	23829740	5.22 × 10^−5^	0.322	0.323			
	2	4054094				1.84 × 10^−6^	0.111	0.093
	3	33737410				3.27 × 10^−7^	0.060	0.107
	5	7996572				1.96 × 10^−5^	0.057	0.073
PTemp	1	2609442				2.80 × 10^−6^	0.084	0.055
	4	33873772				5.72 × 10^−6^	0.051	0.052
	6	6722646	1.29 × 10^−7^	0.356	0.295			
	6	6747298				3.71 × 10^−6^	0.482	0.054
	9	8085354				1.09 × 10^−5^	0.090	0.049

a: MAF: minor allele frequency; b: R square was calculated by R square of model with SNP minus R square of model without SNP

### Whole panel

#### AAC and PRO

Only one locus (chr.6: 1765761) for AAC was detected on chromosome 6. Two loci (chr.5: 13312843; chr.11: 22562237) for PRO were detected on chromosomes 5 and 11, accounting for 15.3 and 12.4 % PVE, respectively (Table 2).

#### PV, HPV, CPV, BD, SB, and CS

Two, four, three, three, four, and three loci were detected for PV, HPV, CPV, BD, SB, and CS, respectively. The major locus (chr.6: 1765761) was detected for PV, BD, SB, and CS, and the nearby locus (chr.6: 1750498) was the most significant site that linked with HPV and CPV. One locus (chr.5: 7756614) for PV was close to the locus (chr.5: 7944999) for HPV. One locus (chr.1: 31640680) for CPV was close to the locus (chr.1: 30634461) for CS. One locus (chr.9: 17538294) for HPV was close to one locus (chr.9: 17539188) for CPV. One locus (chr.7: 1967427) was detected for both BD and SB. One (chr.7: 26540950) for BD and one (chr.7: 25326697) for SB were close to each other (Table 2).

#### PeT and PTemp

A total of five loci located on chromosomes 1, 2, 5, 6, and 8, were detected for PeT. Two loci (chr.6: 7002095; chr.12: 17937512) for PTemp on chromosomes 6 and 12, accounted for 6.5 and 4.1 % of PVE, respectively (Table 2).

### Panel 1 and Panel 2

#### AAC and PRO

One and two loci for AAC were detected in Panel 1 and Panel 2, respectively. The locus (chr.6: 1765761) and locus (chr.6: 1585864) were major QTLs for AAC in Panel1 and Panel 2, respectively. Another locus (chr.9:19310549) for AAC was detected only in Panel 2. Two loci for PRO, located on chromosomes 5 and 11, were identified in Panel 1. No locus was detected for PRO in Panel 2 (Table 3).

#### PV, HPV, CPV, BD, SB, and CS

In Panel 1, GWAS detected 2, 3, 4, 1, 1, and 4 loci for PV, HPV, CPV, BD, SB, and CS, respectively. Similar to the whole panel GWAS results, the locus (chr.6: 1765761) had major effects on BD, and SB, and CS, the locus (chr.6: 1750498) was detected for HPV and CPV, whereas the locus (chr.6: 1666386) was the most significant site for PV. The locus (chr.2: 29640874) for both PV and HPV accounted for 11.8 and 12.1 % PVE, respectively. One locus (chr.9: 15417525) for CPV and one (chr.9: 15474876) for CS was close to each other (Table 3).

In Panel 2, we detected 3, 4, 2, 2, and 1 locus for PV, HPV, CPV, BD, and SB, respectively. No locus was detected for CS. One locus (chr.5: 7944999) was significant for both PV and HPV. One locus (chr.1: 3541593) was detected for both HPV and CPV, while the locus (chr.7: 16503569) for PV was close to one locus (chr.7: 16506178) for both HPV and CPV on chromosome 7 (Table 3).

#### PeT and PTemp

In Panel 1, we detected four loci for PeT on chromosomes 2, 3, 6, and 10, while two loci (chr.6: 6757255 and chr.6: 21712093) for PTemp were detected on chromosome 6, accounting for 21.7 and 15.5 % PVE, respectively (Table 3).

In Panel 2, only one locus (chr.2: 24259382) for PeT and one locus (chr.8: 9459681) for PTemp were detected on chromosomes 2 and 8, respectively (Table 3).

### *indica* and *japonica* panels

#### AAC and PRO

One locus (chr.6: 1529682) and one (chr.6: 1765761) for AAC were detected in *indica* and *japonica* panels, respectively. Three and six loci for PRO were detected in *indica* and *japonica* panels, respectively. One locus (chr.11: 22240707) for PRO in *indica* panel was close to the locus (chr.11: 22563596) detected in *japonica* panel (Table 4).

#### PV, HPV, CPV, BD, SB, and CS

In *indica* panel, one locus for PV, three for HPV, one for CPV, three for BD, and three for SB were detected and no locus was detected for CS. The locus (chr.6: 1750498) was a major QTL for both HPV and CPV, whereas loci for PV (chr.6: 1665483), BD (chr.6: 1721792), and SB (chr.6: 1617418) were close to each other in the *indica* panel. One locus (chr.9: 18729438) for HPV was close to the locus (chr.9: 18443016) for CPV in *indica* panel. One locus (chr.7: 26540950) for BD was close to the locus (chr.7: 25312126) for SB. The QTL (chr.11: 3735389) for BD was close to one QTL (chr.11: 3781133) for SB in the *indica* panel (Table 4).

Three loci for PV, four for HPV, one for CPV, two for BD, three for SB, and two for CS were identified in the *japonica* panel. The QTL (chr.6: 1765761) for PV and SB was close to the locus (chr.6: 1759451) for CS. One locus (chr.9: 18431810) for HPV was close to the locus (chr.9: 18443016) for CPV, one QTL (chr.1: 31774927) for SB was close to one QTL (chr.1: 31668474) for CS in *japonica* panel (Table 4).

#### PeT and PTemp

One locus (chr.1: 23829740) for PeT and one locus (chr.6: 6722646) for PTemp were detected in the *indica* panel, accounting for 32.3 and 29.5 % PVE, respectively.

Three loci for PeT and four for PTemp were detected in the *japonica* panel. The locus (chr.5: 7996572) for PeT were close to the locus detected in the whole panel. The locus (chr.6: 6747298) for PTemp was close to the loci in the whole panel, Panel 1, and the *indica* panel (Table 4).

## Discussion

### Effect of population structure on GWAS

Given that linkage disequilibrium can be caused by the admixture of subpopulations and may result in false-positives if not correctly controlled in the statistical analysis, estimation of population structure must be included in the association analysis [17]. In this study, to determine whether different networks of alleles were associated with ECQ trait variation in the different panels, we performed GWAS on each of the four panels independently and in the panel as a whole. The present study allowed us to compare GWAS results derived from different panels.

The effect of population structure on phenotypic variation was estimated based on analysis of variance (ANOVA) (Table 1 and Additional file 1: Table S1). For the whole panel, eight of 10 traits were strongly affected by population structure. For Panel 1 and 2, nine and five traits were strongly affected by population structure (*P* <0.01), respectively, indicating that Panel 2 (breeding lines) experienced artificial selection, which was less structured than Panel 1. However, only two traits were significantly affected by population structure in the *indica* and *japonica* panels at significance level of *P* <0.01, suggesting that association mapping in the subpopulations, e.g. *indica* or *japonica* subpopulations, with simple population structures can lower false-positives. *indica* and *japonica* varietal groups should be properly treated for association analysis, which may explain why transgressive offspring occur in divergent subpopulation crosses [18] .

### QTL comparison among different panels

For convenience, we regarded those SNPs that were detected in different panels with the physical distance less than 2.0 Mb as the same QTL, and these SNPs were regarded as alleles.

Similar to previous studies, the *Wx* gene is responsible for AAC in these five panels. The single nucleotide polymorphism (SNP) (chr.6: 1765761) for AAC is the *Wx*^*a*^/*Wx*^*b*^ allele (a SNP at the splice site of intron 1), which was present in the whole panel, Panel 1, and *japonica* panel. The *Wx* gene was also the major QTL for AAC in the other two panels, but the *Wx*^*a*^/*Wx*^*b*^ allele was not the most significant association site, suggested that different traits maybe preferred linked with different SNPs. Besides the *Wx* gene, one minor effect locus (chr.9: 19310549) for AAC was detected in Panel 2 (consisted of breeding lines); this locus was close to the QTL flanked by BPH and RM160 [32] (Fig. 3).

One QTL (chr.5: 12559930 ~ 13312843) for PRO detected in the whole panel and Panel 1 was close to the protein content QTL *qPRO5*, which was detected by Xu et al. [8] in the M201-*Wx* subpopulation. The other QTL (chr.11: 22562237 ~ 22563596) for PRO detected in the whole, Panel 2, *indica*, and *japonica* panels was close to *pro11,* which is flanked by RM209 and RM229 [33].

For starch paste viscosity parameters, a specific SNP (chr.6: 1765761), recognized as the *Wx*^*a*^/*Wx*^*b*^ allele, was responsible for 4 (PV, BD, SB, and CS), 3 (BD, SB, and CS), and 2 (PV and SB) in the whole panel, Panel 1, and *japonica* panel, respectively. There were also some SNPs close to *Wx* gene that significantly associated with RVA parameters. However, only one *Wx* gene linked SNP was detected for BD in Panel 2, that may due to the reason that most Korean breeding lines were low-AAC varieties with less diversity. The GWAS results in our study may increase our understanding of the genetic control of starch properties in South Korea *japonica* rice varieties with low AACs and the same *Wx* allele. Besides the *Wx* gene, 12 QTLs were identified in two panels, 3 QTLs were detected in 3 panels, and 2 QTLs were detected in 4 panels (Fig. 3). The QTL (chr.5: 7756614 ~ 8042699) was detected for PV in the whole panel and Panel 2, for HPV in the whole panel, Panel 2 and *japonica* panel, and for PeT in the whole panel and *japonica* panel. This locus has not been detected in previous studies, and is a potentially novel locus specifically present in *japonica* rice varieties. The QTL (chr.9: 17538294 ~ 18443016) for HPV in the whole panel, *indica*, and *japonica* panels, and for CPV in the whole panel and *japonica* panel, was close to *qPV9* for PV, HPV, and CPV [8], which is close to the *isoamylase 3* gene (*Os09g0469400,* chr.9: 17851431 ~ 17868668)*.* The QTL (chr.7: 1118122 ~ 1967427) for BD in the whole panel and *japonica* panel and SB in the whole panel, was close to *qSB7* [7]. The QTL (chr.7: 2532697 ~ 26540950) for BD and SB in the whole panel and *indica* panel was close to the QTL (linked with RM6420) for PV, HPV, CPV, and SB [34], and close to the QTL for SB and CS [8]. The QTL (chr.4: 24056499 ~ 24293919) for SB, detected in the whole panel and Panel 2, was close to the QTL for FV (final viscosity, CPV), PKT, SBV, and TV (HPV), and is linked with RM3785 [35]. The QTL (chr.1: 30627943 ~ 31774927) for CS in the whole panel, Panel 1, and *japonica* panel, and three loci for SB (*japonica* panel), PeT (whole panel), and CPV (whole panel) was 0.21 Mb from the *SSIV-1* gene (*Os01g0720600,* chr.1:30032428 ~ 30041425), indicating that SSIV-1 may influence starch qualities through affecting CPV, SB, CS, and PeT. The QTL (chr.9: 15417525 ~ 15474876) for CS in the whole panel and Panel 1, and for CPV in Panel 1, was close to *qCS9* described by Yan et al. [7]. The QTL (chr.3: 33737410 ~ 33893179) for PeT was detected in Panel 1 and the *japonica* panel, and was close to *Btemp* (peak temperature) flanked by RM203 and RM422 [15]. The QTL (chr.6: 6722646 ~ 7002095) for PTemp, detected in the whole panel, Panel 1, *indica*, and *japonica* panels, was close to the *ALK* gene (*Os06g0229800,* chr.6: 6748398 ~ 6753302) [36], which encodes starch synthase IIa (SSIIa).

### QTLs detected in specific panels

#### Panel 1

The locus (chr.1: 27287504) for HPV, detected only in the Panel 1, was about 1.9 Mb away from the *ADP-glucose pyrophosphorylase large subunit 2* (*AGPL2*) gene (*Os01g0633100,* chr.1:25353805 ~ 25361883). The locus (chr.2: 29640874) for PV and CPV was about 1.6 Mb from the *starch synthase II-2* (*SSII-2* or *SSIIb*) gene (*Os02g0744700*, chr.2:31233292 ~ 31238210). The locus (chr.11: 24255295) for CS was close to *qSB11b* (Yan et al. 2014). The locus (chr.4: 27772775) for CPV was close to the QTL for PV and FV, which is linked with RM255 [35].

#### Panel 2

The QTL (chr.7: 16503569 ~ 16506178) for PV, HPV, and CPV was close to *qHPV7* and *qCPV7*, which are linked with RM418 [37]. The locus (chr.1:3541593) for HPV and CPV, and locus (chr.1: 5102566) for PV were close to the QTL for fa (DP <12) and HPV, which are linked with RM7278 [35]. The locus (chr.2: 8596549) for BD was close to *qBD2* [7]. The locus (chr.2: 24259382) for PeT was close to the *qBTemp2* for peak temperature [7].

##### *indica* panel

The locus (chr.3: 26688241) for HPV was close to the *qHPV3.2* [8].

##### *japonica* panel

The locus (chr.4: 6536977) for PRO was close to the *qPC-4* [38]. The locus (chr.8: 2885700) for PV was close to the *qPV8* [39] and *qPKV8* [7]. The QTL (chr.2: 4054094) for PeT was close to the *qPeT2* [34].

## Conclusions

Overall, we investigated the genetics of 10 rice ECQ parameters by GWAS mapping in the whole panel and four derived panels. GWAS within subspecies panels (*indica/japonica*) cannot only lower false-positives influenced by population structure, but can also increase our understanding of ECQ differences between *indica* and *japonica* subspecies. Comparison of GWAS results indicated that some loci were common in different panels, while some were in specific panels. Loci detected in more than one panel were potentially reliable QTLs for ECQs, and these QTLs should be properly used in molecular breeding to improve rice ECQs, particularly in the Korean *japonica* rice with similar AACs and the same *Wx* allele.

## Methods

### Materials

A total of 227 non-glutinous rice accessions from 295 rice accessions (Additional file 2: Table S2) and their whole genome re-sequencing data [40] were used in this study. Here, we defined the 227 accessions as a whole panel for GWAS, among which the core collection of 110 diverse rice accessions was defined as Panel 1, which comprises 57 Korean accessions and 53 rice accessions collected form 27 countries worldwide. The remaining 117 accessions were considered as Panel 2, which consists of breeding lines, one USDA rice accession, three Japan breeding lines, and 113 Korean breeding lines released between 1967 and 2011. Furthermore, all 227 accessions were divided into the *indica* panel (59) and *japonica* panel (168), since *indica*/*japonica* species contribute the most to the population structure. The whole panel was planted in 2014 during the growing season from early May to September at Kongju National University farm, Yeasan Gun, South Korea. A randomized block design with two replications for each line was used in field experiments.

After being air-dried and stored at room temperature for 3 months, all rice samples were de-hulled to brown rice, then milled into white rice, afterwards ground and passed through a 100-mesh sieve to obtain milled-grain flour. All samples were stored in cooling rooms at 4 °C until use.

### Phenotypic data evaluation

#### AAC and protein content

Determination of AAC was performed using the iodine staining method [41]. The absorbance of the solution was measured at 620 nm against the blank solution with a spectrophotometer. AAC was calculated using a standard curve made from rice samples with known AACs. PRO was measured using the Kjeldahl method with a Kjeltec-Foss 70 2400 Auto analyzer using 5.95 as the nitrogen-to-protein conversion factor.

### RVA profile parameters

The RVA profile of rice flour was determined using a Rapid Visco Analyser (RVA-3, Newport Scientific, Warrie wood, Australia). According to the method of AACC61-02, 3.0 g of rice flour (12 % m. b.) was placed in an aluminum canister, after which 25.0 g of distilled water was added. The heating profile was set as follows: (1) the temperature was held at 50 °C for 1 min; (2) the temperature was linearly ramped up to 95 °C until 4.8 min; (3) the temperature was held at 95 °C until 7.3 min; (4) the temperature was linearly ramped down to 50 °C at 11.1 min; and (5) held at 50 °C until 12.5 min.

Four primary parameters were obtained from the pasting curve: PV, HPV, CPV, and PeT. Three secondary parameters were calculated from the primary parameters: BD (PV minus HPV), SB (CPV minus PV), and CS (CPV minus HPV). The viscosity parameters were measured in RVU. PTemp was determined according to the method proposed by Bao [42].

### Statistical analyses of phenotypic data

Analysis of variance (ANOVA) was performed on the different panels to determine the phenotypic variation and population structure influence on phenotypic variation using the general linear model procedure (PROC GLM) by the SAS program (version 9.3, SAS Institute Inc., Cary, NC). In the model, population structure was set as the fixed independent variable, and 10 phenotypic data points were set as the dependent variables.

### Population structure evaluation and principal component analysis

The genome re-sequencing data of the 227 accessions were collected from the 295 rice whole genome re-sequencing data, which were previously obtained using HiSeq 2000 and HiSeq 2500 [40, 43]. After removing SNPs with a minimum frequency <0.05 by filter alignment using TASSEL 5.0, there were 2,071,925, 1,229,263, 1,563,570, 1,178,226, and 574,169 high-quality SNPs remaining for Panel 1, Panel 2, the whole panel, the *indica* panel, and the *japonica* panel, respectively. Population structure was evaluated by the ADMIXTURE program, which estimates individual ancestry and admixture proportions assuming that *K* populations exist based on a maximum-likelihood method [31]. We ran the program with the script:

admixture --cv genotype.bed *k*

*k* ranged from 1 to 5. Principal component analysis (PCA) of each panel was calculated by TASSEL 5.0 with default parameters by using high-quality SNPs.

### Genome-wide association mapping of five panels

To control for false-positives, the P (PCA) + K model, based on a mixed linear model (MLM), was used for genome-wide association mapping for the five panels by GAPIT software [44]. Here the principal components (PCs) were used to control for population structure in GWAS. We used the default parameters for running the GAPIT software: GAPIT < − myGAPIT(Y = myY, G = myG, PCA.total = 3, Model.selection = TRUE). myY is the phenotype matrix and myG is genotype matrix. SNP sites with the lowest *P* value in the peak region (*P* <10^−4^) were considered significant SNPs (or loci) for phenotypic variation. Otherwise, sites with regions having only one SNP significant were considered false-positives. Loci with a physical distance less than 2.0 Mb were considered the same locus.

## Abbreviations

AAC, apparent amylose content; BD, breakdown; CPV, cold paste viscosity; CS, consistency; ECQ, eating and cooking quality; GWAS, genome-wide association study; HPV, hot paste viscosity; PeT, peak time; PRO, protein content; PTemp, pasting temperature; PV, peak viscosity; PVE, phenotypic variation explanation; QTL, quantitative trait locus; RVA, rapid viscosity analyser; RVU, rapid visco units; SB, setback; SNP, single nucleotide polymorphism; SSRGs, starch synthesis related genes.

## Additional files

Additional file 1:
**Table S1.** Phenotypic variation in different panels and contribution of population structure to phenotypic variation. **Figure S1** Population structure of the whole panel. (DOCX 6297 kb) Additional file 2:
**Table S2.** The sources of the rice varieties used in this study. (DOC 32 kb)
