# Supplementary material for: Genome-wide association study of eating and cooking qualities in different subpopulations of rice (Oryza sativa L.)
Source: BMC Genomics. 2016 Aug 20;17:663. doi: 10.1186/s12864-016-3000-z (PMC4992570; doi:10.1186/s12864-016-3000-z)
Supplement: Additional file 1: — Table S1. Phenotypic variation in different panels and contribution of population structure to phenotypic variation. Figure S1 Population structure of the whole panel. (DOCX 6297 kb) [file 12864_2016_3000_MOESM1_ESM.docx]

**Supplemental Material**

Supplement to: Genome-wide association study of eating and cooking qualities in different subpopulations of rice (Oryza sativa L.). Feifei Xu, Jinsong Bao, Qiang He and Yong-Jin Park.

**Genome-wide association study of eating and cooking qualities in different subpopulations of rice (*Oryza sativa* L.)**

**Table of contents**

1. **Table S1** Phenotypic variation in different panels and population structure (Panel 1 and Panel 2) contribution to phenotypic variation
2. **Figure S1 Population structure of the whole panel.**

**Table S1**

|  |  | AAC (%) | PRO (%) | PV (RVU) | HPV (RVU) | CPV (RVU) | BD (RVU) | SB (RVU) | CS (RVU) | PeT (min) | PTemp (℃) |
| --- | --- | --- | --- | --- | --- | --- | --- | --- | --- | --- | --- |
|  | Means±SD^1^ | 21.3±3.8a^3^ | 8.1±1.5a | 247.1±37.7b | 170.5±28.9b | 275.6±35.2a | 76.6±26.6b | 28.5±36.2a | 104.6±17.9a | 6.31±0.3b | 74.7±3.4a |
|  | Range | 10.7~30.7 | 1.6~14.1 | 155.6~347.8 | 85.0~245.3 | 113.6~371.5 | 24.0~176.0 | -117.1~97.2 | 28.6~170.6 | 3.7~6.9 | 61.8~81.5 |
| Panel 1 | CV^2^ | 17.7 | 17.9 | 15.3 | 16.9 | 12.8 | 34.8 | 127.2 | 17.1 | 3.8 | 4.6 |
|  | R square | 0.574 | 0.184 | 0.154 | 0.160 | 0.151 | 0.049 | 0.181 | 0.330 | 0.225 | 0.138 |
|  | P. value | <.0001 | 0.0003 | 0.0017 | 0.0012 | 0.0019 | 0.2624 | 0.0003 | <.0001 | <.0001 | 0.0039 |
|  | Means±SD^1^ | 16.0±2.3b | 7.5±0.7b | 274.7±18.0a | 181.9±17.1a | 268.0±20.8a | 92.8±18.2a | -6.7±21.4b | 86.1±7.9b | 6.4±0.16a | 70.8±1.4b |
|  | Range | 10.2~27.4 | 5.3~9.5 | 32.3~344.0 | 24.2~238.3 | 59.6~339.2 | 8.4~166.9 | -84.5~63.4 | 35.3~129.3 | 5.9~6.8 | 68.1~77.6 |
| Panel 2 | CV^2^ | 14.6 | 9.9 | 6.6 | 9.4 | 7.8 | 19.6 | -320.8 | 9.2 | 2.5 | 1.9 |
|  | R square | 0.017 | 0.031 | 0.375 | 0.133 | 0.050 | 0.460 | 0.257 | 0.226 | 0.253 | 0.064 |
|  | P. value | 0.390 | 0.179 | <.0001 | 0.023 | 0.256 | <.0001 | 0.001 | 0.001 | <.0001 | 0.026 |

Note: 1. SD: standard deviation; 2. CV: coefficient of variation; 3: different letters mean significant difference between Panel 1 and Panel 2 (*P*<0.05).


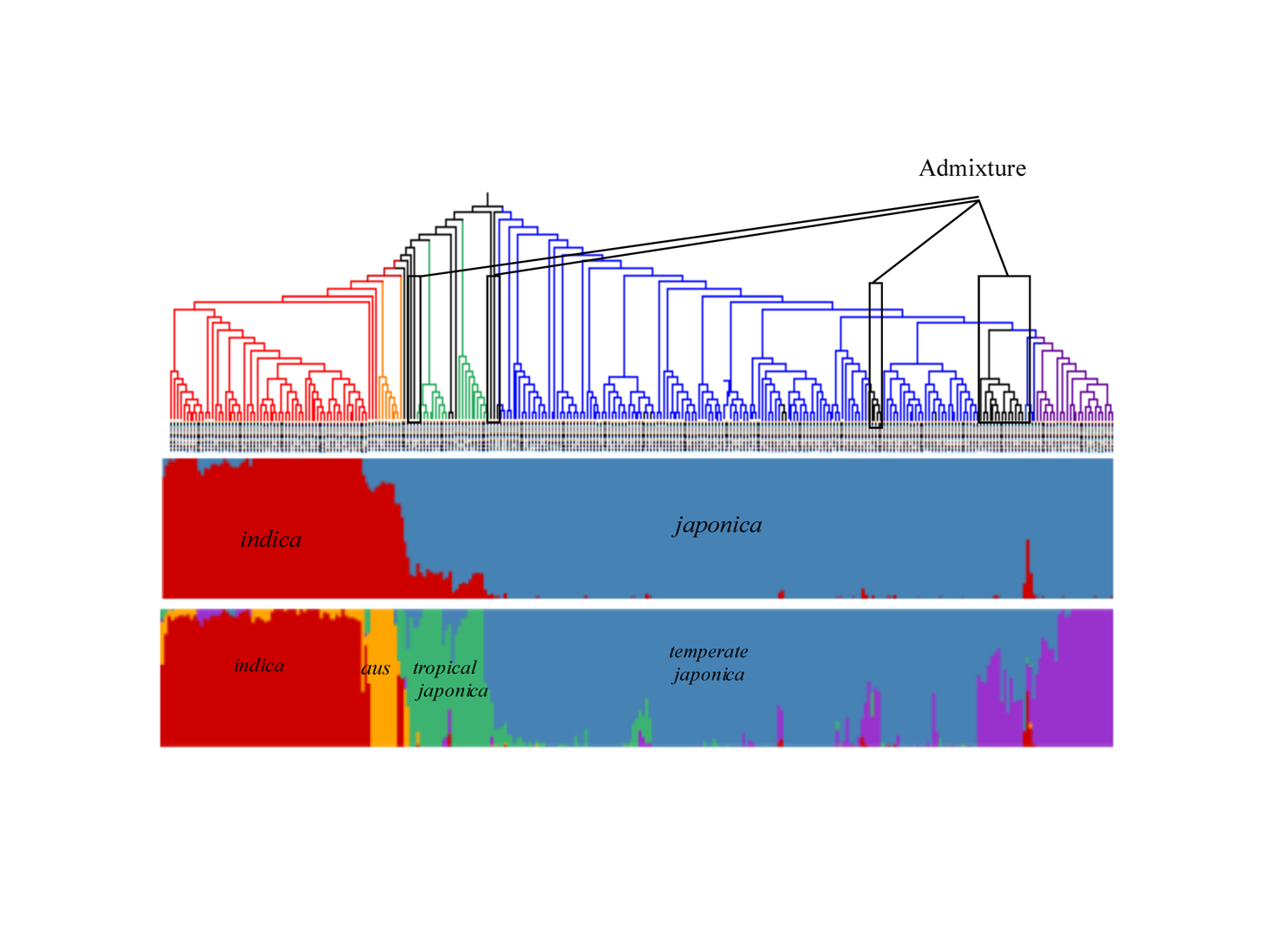


**Figure S1**
