# Supplementary material for: Genome-wide association study of eating and cooking qualities in different subpopulations of rice (Oryza sativa L.)
Source: BMC Genomics. 2016 Aug 20;17:663. doi: 10.1186/s12864-016-3000-z (PMC4992570; doi:10.1186/s12864-016-3000-z)
Supplement: Additional file 2: — Table S2. The sources of the rice varieties used in this study. (DOC 32 kb) [file 12864_2016_3000_MOESM2_ESM.doc]

**Table S2** The sources of rice varieties used in this study.

| Name | English name | Ecotype | Source |
| --- | --- | --- | --- |
| RWG-004 | SUWON 301 | *Temperate japonica* | KOR |
| RWG-005 | Suweon 347 | *Indica* | KOR |
| RWG-006 | CT9993-5-10-1-M | *Indica* | COL |
| RWG-008 | TCHAMPA | *Aus* | IRN |
| RWG-010 | Jejubukjeju-2002-171 | *Indica* | KOR |
| RWG-011 | Jejubukjeju-2002-340 | *Temperate japonica* | KOR |
| RWG-012 | Jejubukjeju-2002-420 | *Temperate japonica* | KOR |
| RWG-013 | Jejubukjeju-2002-521 | *Indica* | KOR |
| RWG-014 | Incheonkanghwasujip-16 | *Temperate japonica* | KOR |
| RWG-015 | Muando | *Temperate japonica* | KOR |
| RWG-016 | Dadajo | *Temperate japonica* | KOR |
| RWG-018 | Sando | *Tropical japonica* | KOR |
| RWG-019 | Batnarak | *Tropical japonica* | KOR |
| RWG-020 | Orido | *Temperate japonica* | KOR |
| RWG-021 | Saducho | *Indica* | KOR |
| RWG-023 | Hanyangjo | *Aus* | KOR |
| RWG-025 | Beobpanhwa | *Temperate japonica* | KOR |
| RWG-028 | YULJOJO | *Temperate japonica* | KOR |
| RWG-029 | Samgyeongjo | *Temperate japonica* | KOR |
| RWG-030 | BAEKGOGNA | *Indica* | KOR |
| RWG-031 | MONDONCHALBYEO(Mongdonjaerae) | *admixture* | KOR |
| RWG-032 | AGBEDE | *Tropical japonica* | NGA |
| RWG-033 | ANBAW C7 | *Temperate japonica* | MMR |
| RWG-034 | BALA | *Indica* | IND |
| RWG-035 | BELLARDONE | *Temperate japonica* | FRA |
| RWG-036 | CHIEM CHANK | *Indica* | VNM |
| RWG-037 | DHARIAL | *Aus* | NPL |
| RWG-038 | DULAR | *Aus* | IND |
| RWG-039 | IR38 | *Indica* | PHL |
| RWG-040 | MAGNOLIA | *Tropical japonica* | USA |
| RWG-041 | MALA | *Indica* | BGD |
| RWG-042 | Mushkan 41 | *Aromatic* | PHL |
| RWG-043 | PUKHI | *Aus* | PAK |
| RWG-044 | Red Rice | *Indica* | IRN |
| RWG-045 | TUN SART | *Tropical japonica* | VNM |
| RWG-046 | VICTORIA F.A | *Temperate japonica* | ARG |
| RWG-047 | Bai Cyue Hwa Lue | *Indica* | TWN |
| RWG-048 | Yangmyeon | *Indica* | TWN |
| RWG-050 | Pyeongbuk 3 | *Temperate japonica* | PRK |
| RWG-051 | YUPUL | *Tropical japonica* | LBR |
| RWG-052 | Liman Belozernij | *Temperate japonica* | RUS |
| RWG-055 | TAI MOCHITO | *Tropical japonica* | THA |
| RWG-056 | WAIKYAKUSHI | *Indica* | TWN |
| RWG-057 | UPLAND | *Tropical japonica* | NGA |
| RWG-058 | NEWREX | *Tropical japonica* | USA |
| RWG-060 | Wu Cyue | *Indica* | TWN |
| RWG-061 | BINATO | *Indica* | PHL |
| RWG-063 | Debzera | *Temperate japonica* | UZB |
| RWG-064 | HAWM SUPAN | *Indica* | THA |
| RWG-065 | Ssal Byeo 22 | *Temperate japonica* | KOR |
| RWG-066 | Urasan | *Tropical japonica* | JPN |
| RWG-067 | XI GUA BAI | *Indica* | CHN |
| RWG-068 | YUNG YUEN CHUEN ZIM | *Indica* | CHN |
| RWG-069 | KENG CHI JU | *Temperate japonica* | CHN |
| RWG-071 | CHIH-TSAO-HE | *Indica* | CHN |
| RWG-072 | HSIANG-HA-TSAN | *Indica* | CHN |
| RWG-074 | Golyeong-2 | *Temperate japonica* | KOR |
| RWG-075 | Golyeong-6 | *Temperate japonica* | KOR |
| RWG-076 | Danyang-7 | *Temperate japonica* | KOR |
| RWG-077 | Danyang-38 | *Temperate japonica* | KOR |
| RWG-078 | Hwaseong-5 | *Temperate japonica* | KOR |
| RWG-079 | Gou 405 | *Temperate japonica* | JPN |
| RWG-080 | Baeksami | *Temperate japonica* | CHN |
| RWG-081 | AKAINE | *Tropical japonica* | JPN |
| RWG-082 | Syarebyeo-61-1-B | *Temperate japonica* | KOR |
| RWG-084 | Syalebyeo-163-1-B | *Temperate japonica* | KOR |
| RWG-085 | Milyang 50 | *Indica* | KOR |
| RWG-087 | Iri 336 | *Temperate japonica* | KOR |
| RWG-088 | Mihyang Byeo | *Temperate japonica* | KOR |
| RWG-089 | MOROBEREKAN | *Tropical japonica* | GIN |
| RWG-091 | Jejubukjeju-2002-550 | *Temperate japonica* | KOR |
| RWG-093 | Huindadak | *Temperate japonica* | KOR |
| RWG-094 | Jotajo | *Temperate japonica* | KOR |
| RWG-095 | Pocheon Jangmang Mebyeo | *Temperate japonica* | KOR |
| RWG-096 | Dongo Byeo | *Temperate japonica* | KOR |
| RWG-097 | Seorianjeunbaengi | *Temperate japonica* | KOR |
| RWG-099 | Jwiippari Byeo | *Temperate japonica* | KOR |
| RWG-100 | Jeongjonghwa | *Temperate japonica* | KOR |
| RWG-101 | Sodujo | *Tropical japonica* | KOR |
| RWG-102 | Sando | *Tropical japonica* | KOR |
| RWG-103 | Bori Byeo | *Temperate japonica* | KOR |
| RWG-104 | Naengjo | *Temperate japonica* | KOR |
| RWG-105 | BIKOM | *admixture* | NGA |
| RWG-106 | British Honduras Creole | *Tropical japonica* | HND |
| RWG-108 | SAHAK | *Aus* | PRI |
| RWG-109 | TAICHUNG-WOO-TSAN | *Indica* | TWN |
| RWG-111 | Xao Bai Mang Sue Dao | *Temperate japonica* | TWN |
| RWG-112 | Di jiao wu jian | *Indica* | TWN |
| RWG-113 | SPIN MERE | *Aus* | AFG |
| RWG-114 | Norin 22 | *Temperate japonica* | JPN |
| RWG-115 | Ssalbyeo 16 | *Temperate japonica* | KOR |
| RWG-116 | NIAN CHI SHI | *Indica* | CHN |
| RWG-118 | TSONG-GAN-SHUN | *Indica* | CHN |
| RWG-120 | YANG-SHA-TSAN | *Indica* | CHN |
| RWG-121 | Chungdo 23 | *Indica* | KOR |
| RWG-122 | Chungdo Hwayang 12 | *Indica* | KOR |
| RWG-123 | Chungdo Hwayang 14 | *Indica* | KOR |
| RWG-124 | Sungju 3 | *Indica* | KOR |
| RWG-125 | Jangsung 1 | *Indica* | KOR |
| RWG-126 | Soonchun 5 | *Indica* | KOR |
| RWG-127 | Daegu Damti 6-2 | *Temperate japonica* | KOR |
| RWG-128 | Guechang 15 | *Temperate japonica* | KOR |
| RWG-129 | Danyang 9 | *Temperate japonica* | KOR |
| RWG-130 | Chungsongaengmi 4 | *Temperate japonica* | KOR |
| RWG-131 | Suhyeonghando | *Indica* | CHN |
| RWG-132 | Xiao zao huang | *Tropical japonica* | CHN |
| RWG-133 | Namseon 34 | *Temperate japonica* | KOR |
| RWG-134 | Namseon 126 | *Temperate japonica* | KOR |
| RWG-135 | Milyang 88 | *Temperate japonica* | KOR |
| RWG-136 | Dudo | *Temperate japonica* | KOR |
| RWG-138 | Jinbu Byeo | *Temperate japonica* | KOR |
| RWG-140 | Hopyung | *Temperate japonica* | KOR |
| RWG-142 | Gopum | *Temperate japonica* | KOR |
| RWG-143 | Unkwang | *Temperate japonica* | KOR |
| RWG-144 | Haiami | *Temperate japonica* | KOR |
| RWG-145 | Samgwang | *Temperate japonica* | KOR |
| RWG-146 | Hopum | *Temperate japonica* | KOR |
| RWG-148 | Ilpum Byeo | *Temperate japonica* | KOR |
| RWG-149 | Chucheong Byeo | *Temperate japonica* | JPN |
| RWG-150 | Junam Byeo | *Temperate japonica* | KOR |
| RWG-151 | Shindongjin Byeo | *Temperate japonica* | KOR |
| RWG-152 | Odae Byeo | *Temperate japonica* | KOR |
| RWG-153 | Nam Il | *Temperate japonica* | KOR |
| RWG-154 | Hwaseong Byeo | *Temperate japonica* | KOR |
| RWG-155 | Boramchan | *Temperate japonica* | KOR |
| RWG-157 | Dongjin Byeo | *Temperate japonica* | KOR |
| RWG-158 | Nakdong Byeo | *Temperate japonica* | KOR |
| RWG-159 | Giho Byeo | *Temperate japonica* | KOR |
| RWG-160 | Hwacheong | *Temperate japonica* | KOR |
| RWG-162 | Chinnong | *Temperate japonica* | KOR |
| RWG-163 | Hanareum | *Indica* | KOR |
| RWG-164 | Dasan Byeo | *Indica* | KOR |
| RWG-165 | Milyang 23 | *Indica* | KOR |
| RWG-166 | Samgang Byeo | *Indica* | KOR |
| RWG-167 | Nokyang | *Tropical japonica* | KOR |
| RWG-168 | Tongil | *Indica* | KOR |
| RWG-171 | Sambaek Byeo | *Temperate japonica* | KOR |
| RWG-172 | Junghwa Byeo | *Temperate japonica* | KOR |
| RWG-173 | Geuru Byeo ? | *Temperate japonica* | KOR |
| RWG-174 | Inwol Byeo | *Temperate japonica* | KOR |
| RWG-175 | Sangmibyeo | *Temperate japonica* | KOR |
| RWG-176 | Geumo Byeo | *Temperate japonica* | KOR |
| RWG-178 | Jinmi Byeo | *Temperate japonica* | KOR |
| RWG-179 | Heugjinju Byeo | *Temperate japonica* | KOR |
| RWG-180 | Jeogjinju Byeo | *Temperate japonica* | KOR |
| RWG-182 | Donghae Byeo | *Temperate japonica* | KOR |
| RWG-183 | Seoan Byeo | *Temperate japonica* | KOR |
| RWG-184 | Gancheok Byeo | *Temperate japonica* | KOR |
| RWG-185 | Juan Byeo | *Temperate japonica* | KOR |
| RWG-186 | Yeonghae Byeo | *Temperate japonica* | KOR |
| RWG-187 | Sobi Byeo | *Temperate japonica* | KOR |
| RWG-188 | Haepyeong Byeo | *Temperate japonica* | KOR |
| RWG-190 | Daelip Byeo 1 | *Temperate japonica* | KOR |
| RWG-192 | Ilmi Byeo | *Temperate japonica* | KOR |
| RWG-193 | Nampyeong Byeo | *Temperate japonica* | KOR |
| RWG-194 | Gyehwa Byeo | *Temperate japonica* | KOR |
| RWG-195 | Yeongnam Byeo | *Temperate japonica* | KOR |
| RWG-196 | Yangjo Byeo | *Temperate japonica* | KOR |
| RWG-198 | Heugnam Byeo | *Temperate japonica* | KOR |
| RWG-199 | Goamy Byeo | *Temperate japonica* | KOR |
| RWG-200 | Manmi | *Temperate japonica* | KOR |
| RWG-201 | Heugkwang Byeo | *Temperate japonica* | KOR |
| RWG-204 | Anda Byeo | *Indica* | KOR |
| RWG-206 | Gaya Byeo | *Indica* | KOR |
| RWG-207 | Baegyang Byeo | *Indica* | KOR |
| RWG-208 | Cheong Cheong Byeo | *Indica* | KOR |
| RWG-213 | Pungmi | *Temperate japonica* | KOR |
| RWG-215 | Hanmaeum | *Temperate japonica* | KOR |
| RWG-216 | Hwasin 1 | *Temperate japonica* | KOR |
| RWG-218 | Gangbaek | *Temperate japonica* | KOR |
| RWG-225 | Danmi | *Temperate japonica* | KOR |
| RWG-227 | Younghojinmi | *Temperate japonica* | KOR |
| RWG-229 | Joun | *Temperate japonica* | KOR |
| RWG-231 | Gangchan | *Temperate japonica* | KOR |
| RWG-232 | Shinbaeg | *Temperate japonica* | KOR |
| RWG-233 | Geongganghongmi | *Temperate japonica* | KOR |
| RWG-234 | Sodami | *Temperate japonica* | KOR |
| RWG-235 | Sukwang | *Temperate japonica* | KOR |
| RWG-236 | Seonhyangheukmi | *Temperate japonica* | KOR |
| RWG-237 | Pungok | *Temperate japonica* | KOR |
| RWG-238 | Wase Gingbouzu | *Indica* | USDA |
| RWG-239 | Palgoeng | *Temperate japonica* | KOR |
| RWG-241 | Milseong | *Temperate japonica* | KOR |
| RWG-242 | Yusin | *Indica* | KOR |
| RWG-243 | Satbyeol Byeo | *Indica* | KOR |
| RWG-244 | Milyang 42 | *Indica* | KOR |
| RWG-245 | Dobong | *Temperate japonica* | KOR |
| RWG-248 | Seomjin Byeo | *Temperate japonica* | KOR |
| RWG-249 | Youngdeok | *Temperate japonica* | KOR |
| RWG-250 | Seohae | *Temperate japonica* | KOR |
| RWG-251 | Mimyeon | *Indica* | KOR |
| RWG-252 | MS11 | *Temperate japonica* | KOR |
| RWG-253 | KOSHIHIKARI | *Temperate japonica* | JPN |
| RWG-254 | Sobaeg Byeo | *Temperate japonica* | KOR |
| RWG-255 | Sangju Byeo | *Temperate japonica* | KOR |
| RWG-256 | Samcheon Byeo | *Temperate japonica* | KOR |
| RWG-257 | Munjang Byeo | *Temperate japonica* | KOR |
| RWG-260 | Saesangju | *Temperate japonica* | KOR |
| RWG-261 | Manchu Byeo | *Temperate japonica* | KOR |
| RWG-262 | Nongan Byeo | *Temperate japonica* | KOR |
| RWG-263 | Sura Byeo | *Temperate japonica* | KOR |
| RWG-264 | Bonggwang | *Temperate japonica* | JPN |
| RWG-266 | Dongan Byeo | *Temperate japonica* | KOR |
| RWG-267 | Daesan Byeo | *Temperate japonica* | KOR |
| RWG-268 | Nongho Byeo | *Temperate japonica* | KOR |
| RWG-269 | Manguem Byeo | *Temperate japonica* | KOR |
| RWG-270 | Saegyehwa | *Temperate japonica* | KOR |
| RWG-272 | Hyangmi Byeo 1 | *Indica* | KOR |
| RWG-273 | Hwangkeumbora | *Temperate japonica* | KOR |
| RWG-274 | Cheonga | *Temperate japonica* | KOR |
| RWG-275 | Cheongdam | *Temperate japonica* | KOR |
| RWG-276 | Keunseom | *Indica* | KOR |
| RWG-278 | Hwanggeumnodeul | *Temperate japonica* | KOR |
| RWG-279 | Cheongan | *Temperate japonica* | KOR |
| RWG-280 | Deuraechan | *Temperate japonica* | KOR |
| RWG-281 | Jinbaek | *Temperate japonica* | KOR |
| RWG-284 | Dongbo | *Temperate japonica* | KOR |
| RWG-285 | Seolemi | *Temperate japonica* | KOR |
| RWG-286 | Jungsaenggold | *Temperate japonica* | KOR |
| RWG-287 | Saeilmi | *Temperate japonica* | KOR |
| RWG-288 | Seokwang | *Temperate japonica* | KOR |
| RWG-289 | Saenara | *Temperate japonica* | KOR |
| RWG-290 | Paldal | *Temperate japonica* | KOR |
| RWG-292 | Chupung Byeo | *Indica* | KOR |
| RWG-293 | Kwanak Byeo | *Temperate japonica* | KOR |
| RWG-294 | Seonam Byeo | *Temperate japonica* | KOR |
| RWG-295 | Daw Dam | *Tropical japonica* | KOR |
